# Empowering Non-healthcare Students as Simulation Assistants in the Digital Era of Simulation-Based Healthcare Education: Bridging the Gap

**DOI:** 10.7759/cureus.50083

**Published:** 2023-12-06

**Authors:** Refka Al-Bayati, Krystina M Clarke, Julia Micallef, Carol D Rodgers, Adam Dubrowski

**Affiliations:** 1 Health Sciences, Ontario Tech University, Oshawa, CAN

**Keywords:** simulation technology, skills and simulation training, health science, healthcare shortages, educational pathways, simulation technicians, healthcare education, simulation-based education, simulation in healthcare

## Abstract

Simulation-based education plays a pivotal role in various high-stakes fields, notably in healthcare, where simulation technicians are crucial for the effective operation of simulation technology. Currently, these roles are often filled by healthcare professionals who transition from patient care, exacerbating shortages in the healthcare workforce. This editorial addresses the current gap by proposing an alternative solution, creating educational pathways for undergraduate students in science and health science programs to become "simulation assistants". Leveraging their foundational knowledge in biological and physical sciences, research skills, and attributes developed through health sciences programs, these students could support simulation activities while entering an ever-evolving field with copious growth opportunities. Paralleling the historical development of medical laboratory sciences, which saw the creation of distinct roles for technologists and assistants, the editorial suggests a collaborative model wherein simulation technicians and assistants work together to enhance simulation-based education in the healthcare sector. This paradigm shift has the potential to alleviate the growing healthcare personnel shortages. While acknowledging the challenges, the editorial envisions the transformative impact of integrating simulation assistants into the healthcare workforce, echoing the historical evolution of specialized roles in response to the changing demands of healthcare.

## Editorial

Simulation-based education serves as a valuable tool across various high-stakes fields, providing trainees with a secure environment to hone their skills without any risk of harm. In recent decades, the healthcare sector has seen an increasing integration of simulation-based learning. This method aims to teach by replicating real-life medical scenarios using various techniques, ranging from the commonly seen silicon suture kits to high-fidelity manikins to cutting-edge virtual reality surgical simulation. Within this context, professionals known as "simulation technicians" also called simulation specialists specialize in the operation and maintenance of simulation technology. They are primarily tasked with ensuring the smooth and efficient operation of simulation equipment and scenarios to optimize training outcomes [1]. Simulation centers and teaching hospitals typically rely on existing personnel with healthcare backgrounds who have transitioned into simulation technician roles during their career journey, attributing their simulation expertise to prior hands-on experience supplemented with simulation-specific courses and certifications [1,2]. As the number of institutions continues to rise, with over a thousand simulation centers across the US and Canada (http://surl.li/nvlmx), and with the emergence of increasingly advanced technological methods, there arises a corresponding demand for personnel possessing elevated technical skills aid in facilitating them. 

Current gap

Currently, simulation jobs, such as simulation technicians, are retained by healthcare providers such as nurses, paramedics, and respiratory therapists who have abandoned their original roles and undergone retraining [2]. This exacerbates the strain on the already understaffed healthcare system. Concomitantly, the demand for more regulation of simulation technicians has led to the development of competency frameworks [3], various graduate programs (http://surl.li/lnjqk), and certification exams such as the one offered by the Society of Simulation in Healthcare (http://surl.li/lnjqp). Notably, the majority of programs offered are at the master’s level, and the competencies that have been established [3] primarily pertain to individuals who have acquired both post-secondary education and practical experience in the healthcare sector. This leaves a gap where only personnel with a higher level of healthcare education can assume this role. This migration of highly educated and skilled healthcare workers from real to simulated patients may harm the healthcare system and underscores the possible need for alternative, structured career pathways training in simulation.

Solution 

In this editorial, we propose a way to close the identified gap by looking at a different population of learners who may be woven into the fabric of personnel to support simulation-based education. More specifically, we suggest creating educational pathways for undergraduate students of Bachelor of Sciences and Health Sciences programs to assume roles as simulation assistants [1]. 

The skills that students in a Bachelor of Sciences and Health Sciences program typically acquire vary depending on contextual factors including the country, university, specific program, and concentration within the field. However, there are some common competencies that students in such programs develop which may provide strong foundations to support simulation-based education [4]. For example, skill- and knowledge-based competencies that these students gain during their education form strong foundations in biological and physical sciences including human anatomy, physiology, microbiology, chemistry, and physics. Equipped with these foundations, these students may have sufficient skills and knowledge to support simulation activities such as simulation development and preparation and high-fidelity scenario execution. In addition, many health sciences programs also include coursework in research methods and statistics to prepare students for conducting and evaluating scientific research in the field. These skills can be easily applied to scholarship of teaching and learning in simulation which may include program evaluation, economics, and educational quality improvement. Finally, the unique skills and knowledge about ergonomics and human factors as well as healthcare systems and policy - all of which fall within the domain of healthcare science programs - may provide new and exciting roles that simulation plays in healthcare. 

In addition to skills and knowledge-based education, health sciences programs also foster the development of various attributes that are valuable for personal, professional, and societal growth, which are important to support the delivery of simulation-based education in healthcare. For example, students learn to critically evaluate scientific literature, healthcare policies, and patient data to make informed decisions and solve complex problems. In many programs, and areas of specialization, effective communication with patients, colleagues, and other healthcare professionals is essential, where students practice both written and verbal communication skills. Lastly, collaboration with healthcare teams; high standards of professionalism including ethical behavior, punctuality, and a commitment to patient care; and leadership skills are cornerstones of most health science programs, which could be critical for those who may specialize in the area of healthcare simulation. 

A model to follow

The original goal of this editorial was to bring to the simulation community’s attention a potential gap and provide a feasible solution. The identified gap is the drain on the healthcare system, where trained healthcare professionals with significant experience in their field are retraining as simulation technicians. We propose a solution to address this gap by creating novel educational pathways for undergraduate students in science and health science programs. This solution may require an initial reconceptualization of what the simulation technician’s job is and augment current taxonomies and competencies frameworks to reflect this. Although daunting, history shows that as healthcare fields evolve, new jobs are created, and new educational pathways need to be built to address these changes. For instance, the evolution of the field of medical laboratory sciences saw workforce needs to evolve and develop, leading to the creation of new jobs, job trajectories (Medical Laboratory Technologists and Medical Laboratory Assistants), and educational pathways at both the college and university level to support training and the growing needs of the profession. 

A review of this history may be fruitful in the development of a model for the simulation community to follow. The emergence of medical laboratory sciences can be traced back to the rapid advancements in medical science and technology during the 20th century. This evolution was driven by the growing demand for skilled professionals who could support healthcare providers in the critical task of diagnosing and monitoring diseases through laboratory testing. Historically, medical laboratory work was often learned through apprenticeships or on-the-job training. However, as healthcare became more complex and reliant on laboratory tests, the need for standardized education and training became evident [5].

The transformation began with the establishment of formal education programs in medical technology during the mid-20th century, such as the one at the Mayo Clinic in Rochester, Minnesota, which was established in 1917. These programs aimed to provide students with a comprehensive understanding of laboratory techniques, equipment, and the scientific principles behind diagnostic testing. The graduates of these programs joined the workforce as Medical Laboratory Technologists. 

As medical science continued to advance, the demand for laboratory personnel expanded. To meet this demand, educational institutions developed dedicated Medical Laboratory Assistant programs. These programs focused on training individuals in phlebotomy, specimen accessioning and processing, and routine equipment maintenance. They also emphasized the importance of ethics, safety, and patient confidentiality in laboratory settings.

In general, Medical Laboratory Technologists and Medical Laboratory Assistants have distinct job roles in healthcare. Technologists, with at least a bachelor's degree, conduct complex diagnostic tests, analyze data, interpret results, and ensure quality control. They play a critical role in disease diagnosis and treatment planning, often collaborating directly with physicians and nurses. In contrast, Assistants, typically holding a certificate or associate degree, focus on specimen collection, preparation, and transportation. They also handle routine tasks, maintain equipment, and manage inventory. Medical Laboratory Technologists and Medical Laboratory Assistants work together to ensure the smooth operation of the clinical laboratory, with Medical Laboratory Assistants providing valuable assistance to Medical Laboratory Technologists in the pre-analytical phase of testing, helping provide quality healthcare where no corners are cut (http://surl.li/lnkhw).

By drawing a parallel between the progression of medical laboratory sciences and the corresponding workforce requirements, we can envision innovative solutions. This editorial does not advocate for replacing the invaluable simulation technicians entrenched in the healthcare industry. Instead, it proposes augmenting the job market with simulation assistants-proficient experts well-versed in educational theory, technology, and scholarly practices to collaborate with simulation technicians. Similar to the collaborative efforts of Medical Laboratory Technologists and Laboratory Assistants, a partnership between a simulation technician and a simulation assistant could yield unique and complementary contributions to the expanding realm of simulation-based education. Such a shift could potentially grant healthcare professionals more opportunities to apply their skills to direct patient care, ultimately alleviating personnel shortages within the healthcare system.

Future directions

This editorial’s purpose was to identify and address a gap in the training of simulationists by proposing an educational pathway for undergraduate science and health science program students to become "simulation assistants”. As such, the aim was to articulate a clear and well-defined position on this topic and contribute a unique or novel perspective to the existing body of knowledge in the field. Furthermore, our intent was not to provide an in-depth analysis of the issue, as this, along with the design, piloting, evaluation, and implementation of the proposed program will be described in subsequent publications. This is in alignment with the Medical Research Council Framework for Evaluating Complex Interventions, which will be the guiding methodological framework used as we embark on this research program. Thus, our approach will unfold through a systematic series of steps. Firstly, we will define the program’s theory and curriculum by conducting an exploratory review. Simultaneously, a thorough examination will be undertaken to delineate the requisite competencies for simulation assistants. This will involve synthesizing existing competency frameworks for simulation technicians and refining them through iterative consensus-building activities. The outcome of this process will be the establishment of a robust competency framework tailored specifically for simulation assistants. Building upon this foundation, a curriculum will be crafted, featuring online courses structured to facilitate adaptable and flexible learning. Next, we will conduct interviews with faculty members, collaborating institutions, and representatives from regulatory bodies. The objective of this will be to assess the best steps to seamlessly integrate the proposed program into the existing system, thereby validating its acceptability and feasibility. This validation process will set the stage for an implementation and evaluation phase, wherein the program will be rolled out in carefully planned phases, allowing for iterative adjustments and improvements based on real-world feedback and outcomes. Thus, the current editorial’s intent is not to provide an in-depth analysis of the problem or solution, but instead, it is to highlight an overlooked problem, and provide a clear position on what could be done to address it. 

Conclusion 

Simulation is an emerging facet within the healthcare sector. As it continues to advance and the demands on the workforce shift, as described in this editorial, it bears resemblance to the way medical laboratory sciences emerged to address the growing intricacies of healthcare. This emergence gave rise to specialized groups of professionals, subsequently opening up new avenues for educational routes and employment. Similarly, in simulation, the revisioning of the human capital needed for the operation and maintenance of simulation technology holds the potential to reshape the landscape of healthcare employment and introduce fresh educational avenues for students pursuing science and health sciences education.

Although the journey may be long and demanding, we believe that the potential benefits to the healthcare system as well as the creation of new job opportunities in the healthcare field would make this journey immensely rewarding. 

## References

[1] Bailey R, Taylor RG, FitzGerald MR, Kerrey BT, LeMaster T, Geis GL (2015). Defining the simulation technician role: results of a survey-based study. Simul Healthc.

[2] Lowther M, Armstrong B (2023). Roles and responsibilities of a simulation technician. StatPearls [Internet].

[3] Roche AF, Condron CM, Eppich WJ, O'Connor PE (2023). A mixed methods study identifying the competencies of health care simulation technicians. Simul Healthc.

[4] Frank JR, Snell L, Sherbino J (2015). CanMEDS 2015 Physician Competency Framework. CanMEDS.

[5] Kotlarz VR (1998). Tracing our roots: origins of clinical laboratory science. Clin Lab Sci.

